# Tensions between Western and Indigenous worldviews in pharmacy education and practice: Part III

**DOI:** 10.1177/17151635231202982

**Published:** 2023-10-13

**Authors:** Jaris Swidrovich

**Affiliations:** Leslie Dan Faculty of Pharmacy, University of Toronto, Toronto, Ontario

## Introduction

This article is the final of 3 in a series about the tensions between Western and Indigenous worldviews in pharmacy education and practice. We first explored the general differences between Western knowledge systems and Indigenous ways of thinking, being and doing and how their worldviews, while fundamentally different, may come together. Second, we interrogated evidence-based medicine and what constitutes evidence and truth, followed by an examination of the compartmentalization of knowledge, health and wellness in pharmacy education and practice. It is clear there are central differences between Western and Indigenous worldviews in pharmacy, which are likely to not only affect the experiences of Indigenous Peoples within the pharmacy discipline but also Indigenous Peoples at large. As we explore the third of 3 fundamental tensions—the exclusion of land, place and spirituality in pharmacy education and practice—it becomes clearer that these 3 identified tensions are simply an introduction to the plethora of Western and Indigenous worldview differences in pharmacy education and practice. Fundamentally, *everything we know and have is from the land. After our physical time on Earth ends, we become the land ourselves.* Let’s explore this concept further in the context of pharmacy education and practice.

## Tension #3 in pharmacy education and practice: Exclusion of land, place and spirituality

While similar to the second tension examined, the exclusion of land, place and spirituality in Eurocentric Western-based science and pharmacy education is worthy of its own evaluation. Within the confines and “objectivity” of the Western scientific method, the roles of land, place and spirituality are positioned as untouchable and subjective addendums to the teaching and learning of the health, wellness, disease processes and healing captured in pharmacy education and practice. Meyer^1^ referred to such subjectivity as “the stain Science and research has not yet been able to wash away.” Regarding spirituality, specifically, Meyer^1^ suggested “the topic of spirituality has become a pink crystal New Age embarrassment to all forms of science.” Meyer^1^ used what she refers to as “holographic epistemology” to design a “(k)new understanding of the philosophy of knowledge, inclusive of all 3 aspects of nature: physical, mental and spiritual.” The inclusion of spirituality in Western-science-based professional programs like pharmacy is, as Meyer^1^ described, “not a religious idea, *we just think it is*.” Spirituality, as both a concept and as a word, does not even appear in the national learning outcomes nor accreditation standards for pharmacy education in Canada.^2,3^ The exclusion of spirituality from pharmacy education creates a significant tension for Indigenous learners who operate with the understanding that “we are more than our bodies, more than our minds,” and believe that “matter is not separate from spirit.”^1^

Indeed, Indigenous Peoples’ connection to land is deeply spiritual as well as legally recognized and constitutionally protected in Canada. Through Indigenous teachings and philosophy, we belong to and must be stewards of the land. Colonialism imposed the concept of land ownership whereby the land is separated and owned by a person, people or other entity. Traditional knowledge, languages, cultural practices and oral traditions have been developed for millennia and are all connected to the land.^4^ When this connection is broken, such as through dispossession from colonialism and other assimilationist policies and practices, the health and well-being of Indigenous Peoples is dramatically affected. As such, the ongoing colonial occupation of what is now called Canada translates to disruption of Indigenous Peoples’ connection to the land and therefore continues to affect their health and wellness. When this reality is not taught, learned and fully realized in pharmacy education and practice, we risk being left with the false assumption that the state of Indigenous Peoples’ health in Canada is the result of the choices and realities of individual people and not because of past and current policies and practices of colonialism. If and when we misrepresent the determinants of Indigenous Peoples’ health, we in turn engage in the wrong and/or incomplete approach(es) to addressing health and wellness inequities experienced by Indigenous Peoples. Stated another way, the social determinants of health for Indigenous Peoples include the land and colonialism (past, present and intergenerational), and therefore, attempts to improve the health and wellness of Indigenous Peoples must be inclusive of such determinants of Indigenous Peoples’ health.

Deloria and Wildcat^5^ shared critical and insightful remarks about the role(s) of spirituality, land and place when they noted that “a great deal of what we experience cannot be explained within the metaphysics of Western science,” which is certainly outside the realm of what is captured in pharmacy education and practice, too. Despite the intimate connections, relations and dependency on other living beings, land, air and water of the Earth’s biosphere, science, and certainly pharmacy education and practice, fails to adequately capture such relationships.^5^ Western notions of reality and corresponding ideas of knowledge marginalize an entire realm of human experience, such as spirituality, declaring such experiences as unknowable and, consequently, left out of serious consideration.^5^ “Our continued existence as part of the biology of the planet,” as Wildcat described, “is inextricably bound up with the existence and welfare of the other living beings and places of the Earth: beings and places, understood as persons possessing power, not objects.”^5^ Considering such an intimate relation and dependency, on land and place, particularly as it is understood by Indigenous Peoples, representing health, wellness and a variety of disease states in pharmacy education and practice without regard to spirituality, land and place, is entirely non-Indigenous. Science and pharmacy education and practice “anesthetizes its students and practitioners” to the reality of living peoples’ experiences of spirituality.^5^

Western knowledge rests itself on a foundation of *reason* to understand the true nature of the world, yet it also privileges itself as the fiduciary of all knowledge, with the authority to authenticate or invalidate other knowledge (when it gets around to it); however, the roles of spirituality, land and place in health and wellness, particularly in the context of pharmacy education and practice, are simply ignored.^2,3^ Kellert^6^ commented on the importance of connection to nature for children’s intellectual, emotional, social, physical and spiritual development. Indigenous Peoples’ perceptions of the land, as described by Haig-Brown and Hodson,^6,7^ is that “the land . . . is a complex being—a spiritual and material place from which all life springs.” Our Elders have taught us about the role and meaning of the land and the land’s connection to our well-being.^8-10^ In a direct and literal sense of the land’s connection to human health and wellness, we also know that approximately 25% of the drugs prescribed worldwide are derived from plants.^11^ Unfortunately, none of these concepts and realities are mentioned in the educational outcomes and accreditation standards for pharmacy education in Canada.^2,3^

For Indigenous Peoples, land has always been a defining element of Indigenous cultures.^12^ As King described, “land contains the language, the stories and the histories of a people. It provides water, air, shelter and food. Land participates in the ceremonies and the songs. And land is home. Not in an abstract way.”^12^ In a health profession like pharmacy, it is alarming for the irrefutable connections between the land and the health and wellness of individuals and populations to not be required learning, regardless of the inclusion of Indigenous knowledges and perspectives of health and wellness. The teaching and learning about and practice with Indigenous Peoples becomes problematic and incomplete when several of the social determinants of Indigenous Peoples’ health are omitted. This disregard of land, place and spirituality in pharmacy education perpetuates the privileging of Western intellectual traditions and further delegitimizes and erases Indigenous worldviews.

## Conclusion

Naming and evaluating the tensions between Western intellectual traditions and Indigenous worldviews in pharmacy education and practice is not only critical to understanding the dominance of Western paradigm in pharmacy but also to attempt to consider the tensions experienced by Indigenous learners and practitioners in pharmacy and the care and education patients receive from students and graduates of pharmacy. Similar to Aikenhead’s^13^ description of Indigenous students in science courses in primary and secondary school, Indigenous learners in pharmacy are often expected to set aside their Indigenous ways of knowing. When school science does not nurture students’ Indigenous identities or strengthen their resiliency, most students are not inclined to participate or achieve in these courses,^14-18^ which is likely to remain true for Indigenous students in pharmacy. The tensions between Indigenous identities and Western science ideologies can be severe for Indigenous students and practising professionals and often create a sense of feeling unwelcome.^13^ In a program and profession such as pharmacy, the tensions between Indigenous and Western worldviews are likely to also be realized by Indigenous patients and families who receive care and education from students and graduates of pharmacy, whether Indigenous or not.

Scholars such as Armstrong^19^ have suggested that integration of Indigenous ways of knowing and being in the world might minimize the tensions experienced between Western intellectual traditions and Indigenous worldview. Minimizing such tensions might positively contribute to the recruitment and retention of Indigenous people applying to and graduating from health science programs like pharmacy, for which people in Canada have specifically been called to action.^20^ Integrating multiple knowledge systems and worldviews in “evidence-based” health care and education; defragmenting the teaching, learning and clinical approaches to health and wellness; and addressing the importance of land, place and spirituality are expected to ease the tensions experienced between Western intellectual traditions and Indigenous worldviews inherent within pharmacy education and practice. While such tensions can and should be minimized, it is anticipated that they will always exist in pharmacy education and practice; however, like a braid of sweetgrass, some tension is required when multiple strands (of knowledge systems) come together. Perhaps the use of an “emulsifier” may bring together these knowledge systems in places and spaces where they may be in opposition to one another. So, as Hester and Cheney^21^ noted, “as the Euro-American tradition refines its truths, resolving the contradictions by adding more and more exceptions and greater and greater complexity, these truths may eventually more nearly resemble stories.” In the meantime, though, we will keep sharing our stories, sciences and knowledges, just as we have since time immemorial. ■
